# Advancing the Multifaceted Performance of Chemical-Grafted Silicone Rubbers via Molecular Simulation

**DOI:** 10.3390/polym17101308

**Published:** 2025-05-11

**Authors:** Yu Zou, Weifeng Sun

**Affiliations:** 1 College of Computer Science and Technology, Heilongjiang Institute of Technology, Harbin 150050, China; 2 School of Electrical and Electronic Engineering, Nanyang Technological University, Singapore 639798, Singapore

**Keywords:** addition-curing silicone rubber, chemical graft modification, molecular simulation, first-principles calculation

## Abstract

The present study explores and verifies the chemical modifications achieved by grafting 4-formylcyclohexyl heptanoate (FH) and 4-(2,5-dioxopyrrolidin-1-yl) cyclohexane-1-carbaldehyde (CC) onto addition-curing silicone rubber (SiR). These modifications aim to enhance the electrical insulation performance, moisture resistance, and pyrolysis tolerance of the SiR material, thereby improving its suitability for reinforced insulation in power transmission systems. First-principles calculations demonstrate that both the chemical graft modifications can introduce shallow hole traps of 0.3~0.4 eV and deep electron traps of 0.9~1.0 eV into the polymer molecule of addition-curing SiR for inhibiting charge transport and injection. It is indicated from first-principles oxidation reaction pathways that the chemical grafting of FH or CC contributes positively, rather than impacts negatively, to the oxidative stability of addition-curing SiR. We also reveal how the two proposed species of organic molecules as grafting agents can act on modifying water adsorption uptake, heat capacity, molecular thermal vibration, and polymer pyrolysis of the SiR material, which are highly accountable for its resistances to high-temperature electrical breakdown, moisture aging, and thermal spikes of partial discharge. The comprehensive molecular simulations and material calculations demonstrate that both the grafted agents can significantly intensify polymer molecule aggregations, restrain molecular thermal vibrations, and reduce water adsorption uptakes. One of the preferable graft agents (CC) can also considerably improve polymer pyrolysis tolerance, while contributing to improved high-temperature electrical breakdown strength and moisture resistance of addition-curing SiR. This research highlights the significant potential of graft modification in molecular compositions to improve the electrical insulation, moisture resistance, ambient-temperature thermal stability, and pyrolysis tolerance of addition-curing SiR, offering valuable insights to develop competent elastomeric polymer applied for cable accessory insulation.

## 1. Introduction

Silicone rubbers have garnered significant attention in various industries due to their remarkable properties such as flexibility, thermal stability, and electrical insulation capabilities [1]. Among their myriad applications, silicone rubbers are extensively used in power transmission systems for insulation purposes, where they encounter rigorous operational conditions including high temperatures, moisture exposure, and electrical stress [2,3,4]. However, despite the intrinsic advantages, conventional silicone rubbers still face challenges in optimizing performance under such demanding conditions, particularly concerning charge traps, moisture resistance, and thermal stability [5].

In recent years, researchers and engineers have been exploring novel approaches to enhance the performance of silicone rubber insulation materials through chemical modifications [6]. Chemical grafting of polar-group organic molecules onto the silicone rubber matrix has emerged as a promising strategy to address the limitations associated with conventional materials [7]. By introducing tailored molecular structures into the silicone rubber matrix, chemical grafting aims to impart enhanced functionalities such as improved charge trapping ability, moisture resistance, and thermal stability [8,9,10].

One of the critical aspects influencing the electrical insulation performance of silicone rubbers is the presence of charge traps within the polymer matrix. Charge traps, which can be shallow or deep, play a crucial role in inhibiting electron avalanche breakdown and preventing electrical discharge events. In recent years, there has been growing interest in understanding and manipulating the charge trapping properties of silicone rubbers to enhance their breakdown strength and reliability [11,12]. Several studies have investigated the influence of chemical modifications on the formation and distribution of charge traps within silicone rubber matrices [13,14]. For instance, the introduction of polar-group organic molecules via chemical grafting can create localized charge trapping sites, thereby reducing the risk of electrical breakdown in silicone rubbers [13]. Similarly, the importance of controlling the spatial distribution of charge traps is highlighted through precise chemical engineering approaches to improve insulation performance under high electric field conditions [14].

Moisture ingress is a significant concern in silicone rubber insulation materials, particularly in outdoor applications where exposure to humidity and environmental moisture is unavoidable. Moisture absorption not only compromises the dielectric properties of silicone rubbers but also accelerates aging processes, leading to degradation in insulation performance over time. Addressing the challenges associated with moisture ingress is thus crucial for ensuring the long-term reliability and durability of silicone rubber-based insulation systems. Recent research efforts have focused on developing strategies to improve the moisture resistance of silicone rubbers through molecular simulations [15,16]. Chemical grafting of hydrophobic or hydrophilic organic molecules onto the silicone rubber matrix represents a promising approach to mitigate moisture absorption and enhance moisture-aging resistance [17].

In addition to electrical and moisture-related challenges, silicone rubbers used in power transmission systems must also withstand elevated temperatures and thermal stresses encountered during operation. Thermal degradation processes, such as polymer pyrolysis, can significantly affect the insulation performance and mechanical integrity of silicone rubber materials, leading to premature failure and service disruptions. Enhancing the thermal stability of silicone rubbers is therefore essential for ensuring the reliability and longevity of insulation systems in high-temperature environments. Recent advancements in chemical modification techniques offer promising avenues for improving the thermal stability of silicone rubbers [18,19,20]. By grafting heat-resistant organic molecules onto the polymer matrix, researchers aim to enhance the material’s resistance to thermal degradation and pyrolysis [21,22].

Molecular dynamics simulations have been pivotal in advancing our understanding of polymeric materials, from exploring energy accommodation between gases and polymers for ultra-low-thermal-conductivity insulation [23] to delving into the structure, dynamics, and mechanics of elastomeric polymers under diverse conditions [24]. These simulations are crucial for enhancing insulating materials within high-voltage cable systems [25] and for shedding light on the thermodynamic forces driving contact electrification between polymeric materials [26], guiding the molecular design of polymer nanocomposites with supramolecular networks [27], and informing the mechanistic optimization of multifunctional polymers [28]. Meanwhile, first-principles calculations serve as a pivotal tool for design and analysis in the realm of dielectric materials science, underscoring their significance in conjunction with data-centric approaches for material innovation [29]. Concurrently, first-principles electronic structure calculations can also be applied for polymer/metal interfaces to select polymers with specific functional groups for acquiring strong adhesion to metal electrode surfaces in solid polymer electrolytes [30]. Complementing these insights, the efficacy of first-principles modeling has been demonstrated in dissecting thermal transport phenomena within polymer dielectrics [31].

The primary objective of this study is to investigate the molecular mechanisms underlying enhancements in the electrical insulation performance, oxidation stability, thermal stability, moisture resistance, and pyrolysis tolerance of addition-curing silicone rubber through chemical graft modifications. While the two chemical graft agents proposed in the present study exhibit high compatibility with polar polymers like silicone rubber (SiR), to date, no studies have explored chemical graft application in improving the comprehensive performance of SiR materials. Therefore, leveraging molecular simulation techniques, particularly by molecular dynamics simulations and first-principles calculations, we aim to elucidate the fundamental mechanism governing the interaction between the grafted organic molecules and the host SiR. By gaining insights into the molecular-level processes, we seek to rationalize the observed amelioration in charge trap, oxidation reaction, molecular thermal vibration, water diffusion and uptake, and thermal-spiked pyrolysis of addition-curing SiR, thereby offering valuable guidance to the design and development of advanced SiR materials applied for cable accessory insulation in power transmission systems.

The present study explores the use of two chemical reagents, 4-formylcyclohexyl heptanoate (FH) and 4-(2,5-dioxopyrrolidin-1-yl)cyclohexane-1-carbaldehyde (CC), as grafting molecules to modify addition-curing SiR. The synthesis of FH relies on the esterification of benzoic acid with heptanoyl chloride, a reaction that is simple, cost-effective, and scalable due to the availability and affordability of starting materials and the mild conditions that are required. CC, although involving the construction of a pyrrolidone ring, can also be synthesized through straightforward oxidation and cyclization reactions. Both FH and CC contain reactive carbon double bonds that enable direct grafting onto SiR polymer chains during the vulcanization process, thus avoiding additional material preparation process and associated cost. Given their ease of synthesis, low cost, compatibility with SiR, and industrial feasibility, FH and CC are highly promising graft agents to enhance comprehensive properties of addition-curing SiR, offering significant application value and market potential.

## 2. Theoretical Models and Calculation Methodology

### 2.1. Molecular/Material Models and Calculation Schemes

**Figure 1:**
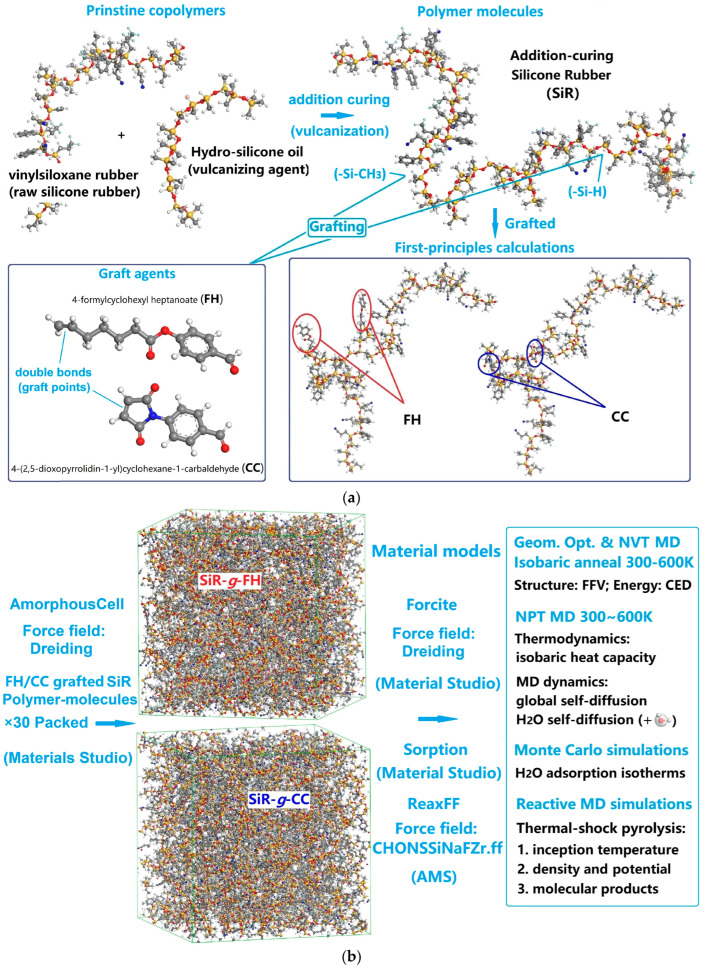
(**a**) Macro-molecule models of chemical-grafted addition-curing SiR polymers for first-principles calculations of electron-states. (**b**) Schematic processes and calculation contents of MD simulations and MC simulations on chemical-grafted addition-curing SiR materials.

Through a complete set of theoretical calculations and molecular or material simulations, including first-principles calculations, Monte Carlo simulations, and normal or reactive molecular dynamics simulations, we have investigated the chemical modifications of grafting two species of organic molecules on the comprehensive properties of addition-curing SiR, as shown in Figure 1. The studied SiR is composed of vinylsiloxane rubber (raw SiR) copolymerized with hydro-silicone oil (addition vulcanizing agent). The degree of polymerization (*D*p) in the polymer molecule model of addition-curing SiR approaches 68 as determined by the number of backbone silicon–oxygen bonds, which is consistent with SiR materials with high transparency and low viscosity applied for cable accessory and electronic packaging (*D*p = 50~100). As shown in Figure 1a, the chemical modifications by grafting organic molecules of 4-formylcyclohexyl heptanoate (FH) and 4-(2,5-dioxopyrrolidin-1-yl) cyclohexane-1 carbaldehyde (CC) separately in ~5 wt% are examined for potentially introducing electronic bound states and electrostatic dipoles, which are highly relevant to charge traps and the thermodynamic properties of SiR material. First-principles calculations of the grafted SiR polymer molecules are performed to demonstrate that grafting FH or CC can introduce deep charge traps to impede charge transports and suppress space charge accumulations.

In constructing the polymer molecule models of chemically grafted addition-curing SiR, the grafting molecules are strategically connected to the reactive groups -Si-CH_3_ and -Si-H located modestly offset from the middle of the polymer chain, as illustrated in Figure 1a. This approach is chosen to more accurately reflect the outcomes of the actual grafting chemical reaction. In addition-curing SiR, the -Si-CH_3_ and -Si-H groups are the most reactive sites that are uniformly distributed along the polymer chain. While the position of the grafting molecules on polymer molecules (e.g., middle or terminal positions) does not significantly influence the electronic structure of polymer molecules or the macroscopic properties of polymer materials, grafting at positions considerably deviating from the exact middle of the addition-curing SiR polymer molecule better captures the interactions between grafting agents and polymer chain segments in actual chemical reactions. In practical chemical synthesis, grafting reactions typically occur at multiple reactive sites rather than being confined to a specific location on the polymer chain. Compared to grafting at terminals or exact middle of polymer chain, grafting at positions properly offset from the middle can reduce steric hindrance to some extent, allowing the grafted polymer chain to maintain a more stable configuration and avoiding local structural distortions caused by concentrated grafting sites.

Multiple-molecule condensed matter (material) models are established through packing 30 polymer molecules of addition-curing SiR grafted with FH or CC molecules into amorphous cells by Monte Carlo (MC) molecular simulations, then relaxing and approaching thermodynamic equilibrium under barostatic atmosphere pressure at various temperatures of 300~600 K through force field geometry optimization and molecular dynamics (MD) simulations, as shown in Figure 1b. Macroscopic properties, such as cohesive energy density, fractional free volume, molecular dynamic self-diffusion, and water (H_2_O) thermodynamic adsorption, of the grafted SiR materials are eventually calculated using these material models, as indicated by the right panel of Figure 1b. The schemes and specifications of MD simulations, MC molecular simulations, and first-principles calculations in the present study, as implemented by the Forcite, AmorphousCell or Sorption, and Dmol3 codes of the Materials Studio software package (Accelrys Inc., Materials Studio version 2020.08, San Diego, CA, USA), respectively, could be found in reference [32].

### 2.2. Oxidation Reaction Pathway

To evaluate the impact of chemical grafting on the oxidative stability of addition-curing SiR, the oxidation reaction barriers (activation energies) and released energies for both the oxidation-vulnerable SiR constituent (hydro-silicone with its terminals being hydrogenated to represent a polymerized molecular chain) and the grafted modifiers (FF and CC with their double bonds being hydrogenated to represent the grafted states) with oxygen molecules are calculated to determine whether the chemical graft modifications affect the oxidative stability of addition-curing SiR. The total energy throughout the oxidation reaction is calculated by the first-principles method as implemented with the Dmol3 code of the Materials Studio package, with similar schemes and specifications to those for calculating the electron states of chemical-grafted SiR polymer molecules. The following steps are taken:

1. Molecular structures of the SiR constituent and the grafted agents are fully optimized to obtain their ground-state geometries.
2. Oxidation reaction pathways are identified by constructing a series of intermediate structures representing the reaction coordinate through transition state searching with the quadratic synchronous transit method, and they are finally refined by the nudged elastic band (NEB) method to provide minimum energy pathways.
3. The energy barrier or released energy is evaluated by determining the energy difference between the reactant and the transition state or product along the reaction pathway.

### 2.3. Reactive Molecular Dynamics Simulation

Reactive MD simulations of NPT ensembles are carried out with the ReaxFF code of the Amsterdam Modeling Suite (AMS) software package (2024.106, Software for Chemistry & Materials BV, Amsterdam, Netherlands) for the pure and grafted SiR materials. The partial discharge (PD) initiated by distorted electric fields in insulation materials used for high-voltage power systems can promptly increase the local temperature to thousands of Kelvin. In addition, it has been demonstrated by reactive MD simulations that a higher specified value of simulation temperature and heat rate result in a higher speed and a lower inception temperature of polymer pyrolysis, respectively, while neither of them evidently accounts for product species and the chemical decomposition reaction path [20,33]. Therefore, to simulate the thermal spike pyrolyses caused by PD’s heating effect in pure and grafted SiR materials, a CHONSSiNaFZr.ff reactive force field (incorporating chemical reactive interactions among carbon, hydrogen, oxygen, nitrogen, sulfur, silicon, sodium, fluorine, and zirconium atoms) and a sharply increasing temperature (heating) rate of 10 K/ps with an objective temperature of 3000 K are specified for describing flash chemical decomposition of SiR polymer chains or grafted molecules under the temperature surge from PD [32].

## 3. Results and Discussion

### 3.1. Charge Traps Introduced by Chemical Grafts

**Figure 2:**
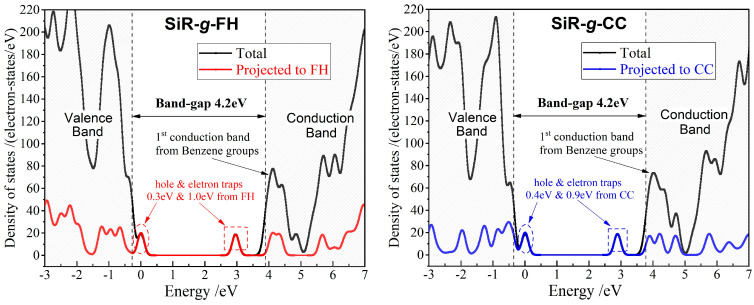
First-principles molecular-projected density of electronic states in chemical-grafted SiR polymer molecules with FH and CC, respectively (SiR-*g*-FH and SiR-*g*-CC).

Electronic properties of addition-curing SiR polymer molecules grafted separately with FH and CC (organic molecules with multiple polar groups) are calculated by the first-principles method, as shown by the energetic distribution spectra of electron states (density of states, DOS) in Figure 2. For polymer dielectrics, a charge trap depth of 0.9 eV is generally used to distinguish between deep and shallow charge traps. According to Boltzmann thermal excitation theory, this charge trap depth corresponds to a thermal excitation temperature of approximately 70 °C, which is the upper operating temperature limit for electrical insulation components in power transmission systems. Therefore, deep traps indicate that the charge trapping mechanism retains effectiveness at temperatures above 70 °C. The deeper the charge trap, the higher the temperature at which it can effectively capture charge carriers.

Both the chemical modifications of grafting FH and CC are competent for introducing shallow hole traps in depths of 0.3~0.4 eV and deep electron charge traps in depths of 0.9~1.0 eV into SiR polymer molecules by rendering occupied and unoccupied electronic bound states just above and far below the valence band maximum (VBM) and conduction band minimum (CBM), respectively. Therefore, the grafted agents present charge traps into addition-curing SiR, which provide local regions of lower electrostatic potentials to increase the rate or probability of scattering charge carriers and thus reduce charge carriers’ mobility or electrical conductivity, as described by the following equations:

***J*** = *γ**E***, *γ* = *nμe*, *μ* = *eτ*/*m*, 1/*τ* = scattering rate (1)

where *μ*, *n*, *e*, *τ*, and *m* denote the mobility, density, charge quantity, scattering time, and effective mass of charge carriers, while ***J*** and *γ* symbolize electric current density and electric conductivity, respectively, and ***E*** represents the electric field. Meanwhile, the grafted FH or CC can inhibit charge injection from electrodes by Coulomb forces after capturing charge carriers (suppressing space charge accumulations) to further improve the electrical insulation performance of addition-curing SiR material [34].

Despite the distinct polar groups and molecular structures of the two graft agents, they both exhibit similar electronic properties when grafted onto SiR polymer chains. Specifically, they both have unoccupied molecular orbitals at 0.9~1.0 eV below the CBM and occupied molecular orbitals at 0.3~0.4 eV above the VBM. The depths of the charge traps introduced by these two chemical graft agents are therefore quite similar. This phenomenon is not coincidental due to the CBM and VBM of addition-curing SiR being considerably higher and lower than the lowest unoccupied molecular orbital energy level and highest occupied molecular orbital energy level of the general organic molecules with polar groups, respectively, as illustrated in Figure 2 by comparing the projected densities of electron states from the grafted agents with that from the host SiR.

### 3.2. Oxidation Stability

**Figure 3:**
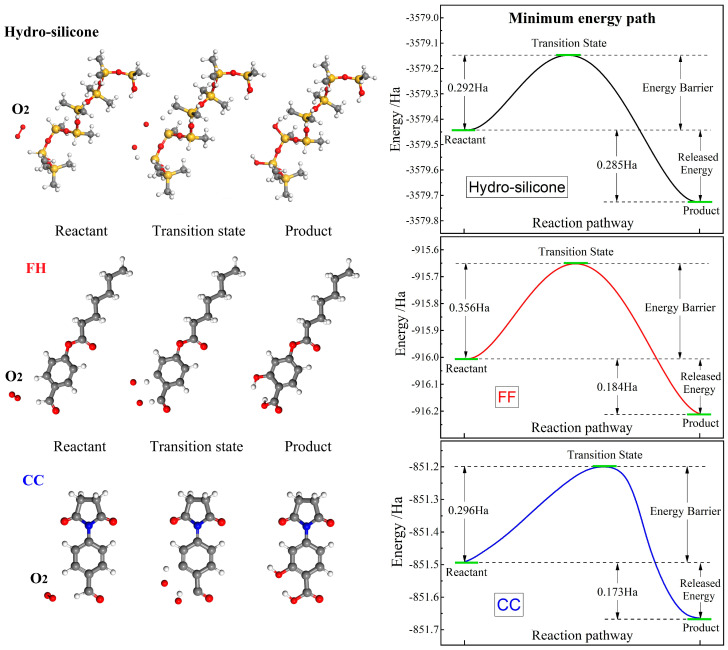
Schematics and minimum energy paths in oxidation reactions of addition-curing SiR constituent (hydro-silicone) and grafted agents (FH and CC) with oxygen molecule.

In the chemical components of addition-curing SiR, the silicon–hydrogen bond (-Si-H) is most susceptible to oxidation under special conditions such as high temperature, ultraviolet irradiation, or the presence of oxygen. During the oxidation process, oxygen reacts with the silicon atom in -Si-H to produce a silanol group (-Si-OH). This hydroxylation reaction disrupts the integrity of SiR molecular chains, leading to performance degradation. In the grafted modifiers proposed in this study, the presence of a benzaldehyde group (-C_6_H_5_CHO) introduces a reactive component that is relatively susceptible to oxidation, producing a benzoic acid group (-C_6_H_5_COOH) through the chemical reaction with the oxygen molecule (O_2_). Therefore, our first-principles calculations of oxidation reactions are specifically focused on the oxidation of -Si-H in addition-curing SiR and -C_6_H_5_CHO in the grafted modifiers. As derived from our first-principles calculations on geometry optimizations of oxidation products, given one oxygen atom in O_2_ reacts specially with carbonyl in -C_6_H_5_CHO in the grafting agents to produce -C_6_H_5_COOH, the other oxygen atom reacts with the adjacent benzene carbon–hydrogen bond (-C-H) and turns into a phenol group (-C-OH), as illustrated in the left panel of Figure 3.

The oxidation stability of a polymer is influenced by the energy barrier and released energy associated with the oxidation reactions of its polymerizing constituent and any grafted modifier. A higher reaction barrier or smaller released energy indicates greater resistance to oxidation, thereby enhancing polymer oxidation stability. The energy barrier and released energy of oxidation reactions for (-Si-H)-containing hydro-silicone as the oxidation-vulnerable constituent in addition-curing SiR are calculated to be 0.292 Ha and 0.285 Ha, respectively, as indicated in the right panels of Figure 3. In comparison, the oxidation reaction barrier and released energy for the grafted agent FH/CC are found to be 0.356/0.296 Ha and 0.184/0.173 Ha, respectively. Both the grafted agents exhibit higher energy barriers and smaller released energies in oxidation reactions compared to hydro-silicone constituent of addition-curing SiR. This suggests that the introduction of these grafting agents does not compromise the oxidation stability of SiR material. In fact, the harder oxidation reactions with oxygen for the grafted agents indicate that they may provide additional resistance to oxidation, thereby enhancing the overall oxidation stability of the grafted SiR materials. This finding is crucial for SiR materials being exposed to oxidative environments where long-term stability and reliability are essential.

### 3.3. Thermal Stability and Moisture Resistance

**Figure 4:**
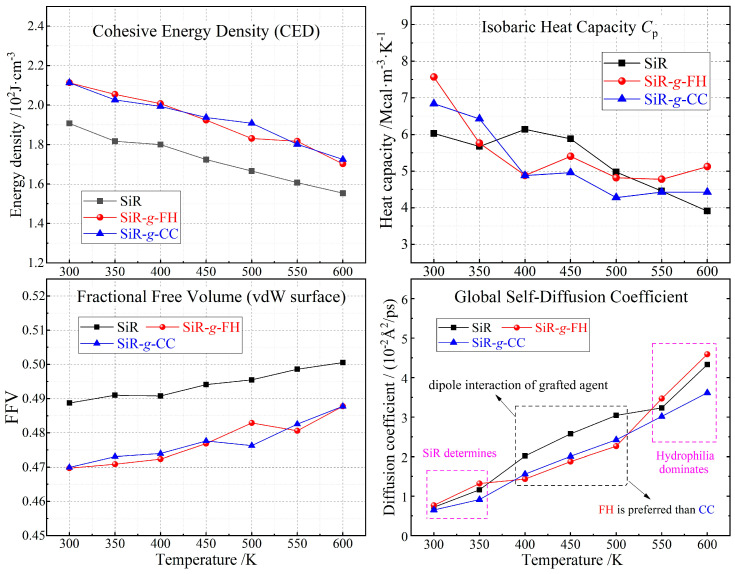
Temperature-dependent profiles of cohesive energy density, fractional free volume, isobaric heat capacity, and global self-diffusion coefficient in addition-curing SiR materials with and without FH or CC graft modification.

The present part is centered around on revealing the modification effects and mechanism on the microstructure, thermal properties, and moisture resistance based on the fractional free volume, cohesive energy density, heat capacity, molecular self-diffusion, and water adsorption uptake of the chemical-grafted addition-curing SiR materials with FH or CC molecules (SiR, SiR-*g*-FH and SiR-*g*-CC) through a series of statistic calculation and comparative analyses on the MD and MC simulation results, as illustrated in Figure 4. Cohesive energy density (CED) is a measure of the energy required to separate close molecules in a cohesive structure due to the van der Waals force between the aggregating molecules and the strength of the hydrogen bonds. For dielectric polymers with polar molecular fragments, the dielectric breakdown strength increases with the increase in the density and strength of polar groups (intrinsic dipoles) in the polymer chains, which is a representation of dipole interaction (dispersion force). Fractional free volume (FFV) describes the amount of unoccupied space or free volume, which is a measure of the mobility or deformation of polymer chains within a polymer material. A lower FFV indicates tighter molecular packing and reduced molecular mobility, which enhances thermal stability and restrains electrothermal breakdown by limiting the expansion and degradation of polymer chains under elevated temperatures. Additionally, reduced FFV minimizes the available space for water molecules to diffuse and adsorb, thereby improving the moisture resistance of polymeric material.

It is reasonable to deduce that the stronger dipole electrostatic forces between polymer molecules in SiR-*g*-FH and SiR-*g*-CC materials than those in pure SiR material, which is derived from additional dipole moments contributed by the grafted agents, leads to a tighter aggregation of SiR molecular chains and therefore a higher thermal stability below 600 K without any conspicuous change in intramolecular chemical bonding forces, which can be verified by the evidently reduced FFV and increased cohesive energy density in a comprehensive temperature range, as indicated by the left panels of Figure 4. Meanwhile, both SiR-*g*-FH and SiR-*g*-CC represent a higher and lower isobaric heat capacity than pure SiR at temperatures below 400 K and above 350 K, respectively, which are beneficial and detrimental to their thermal stability in the ambient-temperature range without pyrolysis of the polymer molecules, as illustrated in the top-right panel of Figure 4. It is eventually confirmed by the weaker molecular thermal vibrations (lower global self-diffusion coefficients) at 400~500 K that both graft-modified SiRs have a higher ambient-temperature thermal stability than pure SiR due to dipole interactions from grafted agents, as indicated by the bottom-right panel in Figure 4.

**Figure 5:**
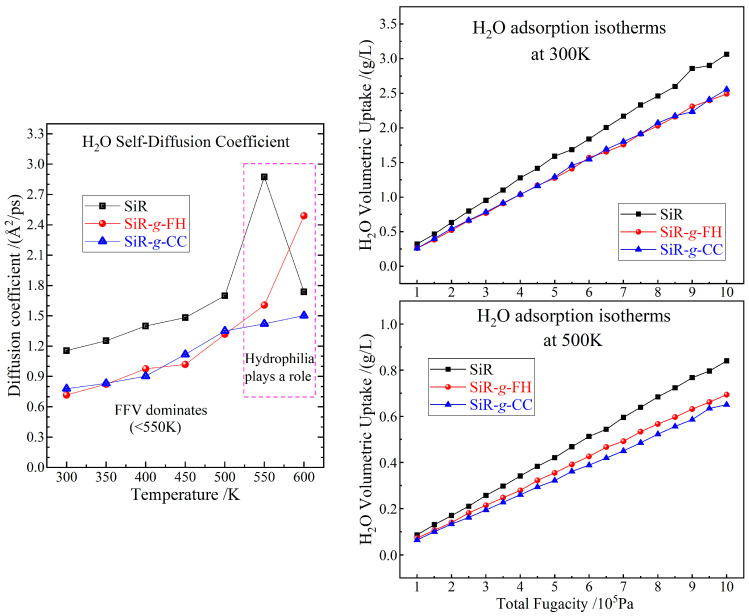
H_2_O self-diffusion coefficient (**left panel**) and adsorption isotherms (**right panels**) in addition-curing SiR materials with and without FH or CC graft modification.

At room temperature, SiR-*g*-FH and SiR-*g*-CC show identical H_2_O uptake, which is significantly less than that of pure SiR due to the graft agent’s stronger hydrophobicity and the grafted material’s smaller FFV, as indicated in the right panels of Figure 5. In contrast, at 500 K, SiR-g-CC exhibits a more evident reduction in H_2_O uptake than SiR-*g*-FH, merely due to the smaller FFV of SiR-*g*-CC than that of SiR-*g*-FH. For polymer materials, the water absorption uptake is determined primarily by both FFV and molecular hydrophilicity/hydrophobicity. Despite the stronger hydrophilicity of the grafted agents, their polar groups render evidently stronger dipole moments than that in the SiR, which leads to much tighter polymer chain aggregations and thus results in a significant reduction in FFV, which dominantly accounts for less H_2_O uptake in the chemical-grafted SiR materials compared with the pure SiR material.

Compared with pure SiR, the slower H_2_O self-diffusion observed in both grafted SiRs at temperatures ranging from 300 to 550 K is primarily attributed to their reduced FFV, as illustrated in the left panel of Figure 5. This lower FFV compresses the available space and channels for water molecules to diffuse, thereby significantly impeding their thermal motions. However, when the temperature exceeds 550 K, the FFV increases substantially, providing ample pathways for water diffusion and effectively eliminating the constraints on thermal motions of water molecules in both pure and grafted SiR materials. As a result, H_2_O self-diffusion becomes dominated by molecular hydrophilicity at 600 K, resulting in slightly faster H_2_O self-diffusion in FH-grafted SiR compared to pure SiR.

Therefore, both the graft-modified SiRs have an enhanced moisture resistance compared with pure SiR in comprehensive temperature ranges due to the dipole interactions from the grafted agents, as indicated by their smaller H_2_O thermodynamic uptakes at 300~500 K and slower H_2_O self-diffusion at 300~550 K due to their lower FFV compared to pure SiR. Overall, the interactions of the chosen organic molecules with the SiR after the grafting process predominantly involve dipole–dipole interactions and hydrogen bonding from every polar group in both the SiR molecular chains and grafted agents, which can thus be highly enhanced by the grafted organic molecules with evidently stronger dipoles than those in the SiR polymer, essentially accounting for the ameliorated properties in chemical-grafted addition-curing SiR materials.

Recent experimental studies have reported that the water uptake of addition-curing SiR is approximately 0.3% at ambient temperature and pressure [35]. These experimental tests are typically conducted on samples that have undergone wet-heat aging, where the water adsorption and diffusion have not yet reached thermodynamic equilibrium under standard conditions. Additionally, the experimental samples may contain impurities that can affect the measured water uptake values. Therefore, the present molecular simulation results unravel that the water uptake of addition-curing SiR at room temperature is in the range of 0.1~0.3% under different pressures, which is consistent with experimental reports and further validates the reliability of the present molecular simulations.

Currently, the primary strategy to enhance the electrical insulation performance of SiR materials is nanocomposite modification with inorganic nanofillers, which generally results in a significant increase in water uptake, with reports indicating an increase of over 20% in water uptake compared to pure SiR [35]. This increase in water uptake will lead to exacerbated thermal–moisture aging, compromising the electrical insulation performance of cable accessory materials in moist environments. In contrast, our study employs a chemical grafting approach to introduce multiple charge traps into addition-curing SiR which effectively inhibit charge transport and injection, thereby enhancing the electrical insulation performance of the SiR material. Importantly, the chemical grafting of FH and CC onto addition-curing SiR significantly enhances moisture resistance while improving electrical insulation performance, which are crucial for the development of advanced SiR materials suitable for high-performance applications in power transmission systems.

### 3.4. Pyrolysis from Thermal Spikes

**Figure 6:**
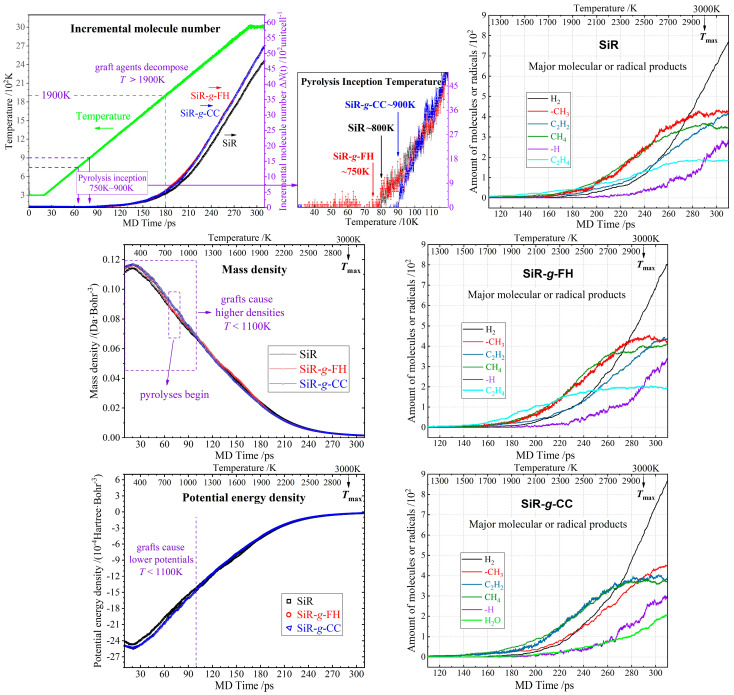
Results from reactive MD simulations on thermal impact pyrolyses of addition-curing SiR materials without (SiR) and with grafting FH (SiR-*g*-FH) or CC (SiR-*g*-CC), as indicated by incremental molecule quantity and MD temperature, mass density, intermolecular potential energy density (**left-column panels**), and major molecular or radical products (**right-column panels**) in thermal spike (rapid isobaric heat rate) processes.

Through reactive MD simulations under a rapid isobaric heat-rating condition, the underlying behavior of the graft modifications on polymer pyrolysis of addition-curing SiR under the thermal impact that may be caused by partial discharge are evaluated to predict whether and how the grafted FH or CC can improve or degrade the SiR’s performance in resisting the thermal effect of partial discharge, as indicated by the complete simulation results in Figure 6. The chemical decomposition of pure and chemical-grafted SiR polymers during a reactive MD heat-rating process is evaluated by the incremental molecular number (the increase in the molecule number in an amorphous unit-cell), mass density, and potential energy density as a function of MD time, as shown by the left panels in Figure 6. In a multi-molecular system, the intermolecular potential energy due to van der Waals forces indicates the strength of molecular aggregation. The density of intermolecular potential energy (here named briefly by potential energy density), particularly in the context of polymer materials, signifies the size or polymerization degree of polymer molecules.

In reference to the pure SiR, the graft-modified SiRs with FH and CC exhibit a reduction and increment in pyrolysis inception temperature by 50 K and 100 K, respectively, at 750~900 K, implying the CC graft modification is more preferable than FH for alleviating the thermal impact pyrolysis of addition-curing SiR. The shift in the pyrolysis temperature below and above the values of pure SiR through grafting FH and CC requires further examination. The FH grafting appears to lower the onset temperature for pyrolysis due to the possible catalytic effects or lower activation energy barriers introduced by the FH molecules. Conversely, CC grafting increases the onset temperature, indicating its effectiveness in enhancing the pyrolysis tolerance of addition-curing SiR. Given no pyrolysis occurs below the inception temperature, both the chemical graft modifications merit enhancing the intermolecular aggregations of SiR polymer chains by significantly contributing dipole interactions, leading to a higher mass density and a lower potential energy density below 1100 K, even when pyrolyses have occurred, which implies a higher thermal stability. When temperature rises above 1900 K, both the grafted FH and CC begin decomposing to produce more pyrolysis products, which nevertheless does not mean they will aggravate the thermal decomposition of the SiR polymer chains, as indicated by the almost equal potential energy densities of the pure and grafted SiRs. These results also qualify the chemical stability of both FH and CC not exacerbating pyrolysis in SiR polymers under the heat effect of PD.

Molecular or radical products of thermal impact pyrolysis in SiR-*g*-CC is distinguished from SiR and SiR-*g*-FH by a considerable amount of H_2_O and almost disappeared ethylene (C_2_H_4_) molecules. Compared to pure SiR, the slightly higher yield merely in hydrogen or methyl as the main pyrolysis products of the two grafted SiRs are very likely to originate from the thermal decomposition of the grafting agent itself. Therefore, it is confirmed that FH and CC molecules possess adequate chemical stability, not exacerbating thermal impact pyrolysis but enhancing the molecular aggregation intensity of SiR polymer molecules by providing much stronger dipole moments, thereby improving both the ambient-temperature thermal stability and high-temperature pyrolysis tolerance of addition-curing SiR material.

Recent experimental studies have demonstrated that the pyrolysis inception temperature of addition-curing SiR is approximately 800 K, which agrees well with the simulation results presented in this study [36,37,38]. These investigations further reveal that incorporating specific chemical additives into the monomers of addition-curing SiR can elevate the onset temperature of pyrolysis by 50 to 150 K. However, such strategies, which involve altering the fundamental polymeric constituents of addition-curing SiR, inevitably lead to significant changes in its electrical properties. Consequently, these approaches are not suitable for enhancing the performance of insulating materials used in cable accessories. In contrast, the present study employs a chemical grafting technique involving a low content of small organic molecules with specific polar groups. This method introduces charge traps that effectively inhibit charge transport and injection within addition-curing SiR while simultaneously enhancing the moisture resistance and pyrolysis tolerance of the SiR material. As a result, this approach reasonably promises significant improvement in the electrical insulation performance of addition-curing SiR material under partial discharge and thermal–humid conditions.

## 4. Conclusions

Through comprehensive molecular simulations and material calculations, chemical modifications by grafting two proposed species of polar-group molecules into addition-curing SiR polymer have been extensively investigated to significantly improve the charge trapping, moisture resistance, and thermal stability of the SiR material. Specifically, these modifications introduce shallow hole traps of 0.3~0.4 eV and deep electron traps of 0.9~1.0 eV, as evidenced by first-principles calculations, being valid to impede charge transports and suppress space charge accumulations. It was verified through reaction transition-state calculations that the grafting agents do not count against but may favor the oxidative stability of addition-curing SiR. Molecular dynamics simulations demonstrate a significant reduction in the atom-averaged self-diffusion coefficient and elevation in cohesive energy density, verifying enhanced polymer aggregation and thermal stability at ambient temperatures. The reduction in water adsorption uptake at both room temperature and 500 K supports improvement in moisture resistance, despite the hydrophilic nature of the grafted agents being counteracted by reduced fractional free volume. The findings underscore the potential of chemical graft modifications to develop advanced SiR materials with improved electrical insulation properties and hydrothermal aging resistance. It was verified by reactive MD simulations that chemical grafting CC is particularly effective at inhibiting thermal impact pyrolysis as caused by partial discharges, increasing the pyrolysis inception temperature by 100 K, without exacerbating polymer decomposition above 1900 K. These insights are critical for guiding the development of SiR materials used in high-performance cable accessories.

The implications of these predicted improvements extend beyond immediate applications in cable insulation. The enhanced charge trapping characteristics suggest potential use in electronic and dielectric applications where the control of charge mobility and injection is crucial. The improved moisture resistance and thermal stability indicate suitability for harsh environmental conditions, expanding the potential for outdoor and automotive applications. The ability to withstand high temperatures without significant degradation opens avenues for use in aerospace and industrial sectors where materials are exposed to extreme thermal stresses. Thus, the chemical graft modifications studied here provide a versatile approach to enhancing the performance of SiR materials in a wide range of advanced technological applications. Nevertheless, due to the limitations in the ground state method of first-principles calculations and the classic force field method of the molecular dynamics simulations utilized in the present study, we have not rendered any direct evidence on how charge traps introduced by chemical grafts affect the macroscopic electrical properties, such as dielectric permittivity, electric resistivity, and dielectric breakdown strength, etc., of the SiR material, which are expected to be fulfilled in future studies by molecular dynamics ab initio calculations and experimental validations.

## Data Availability

The raw data supporting the conclusions of this article will be made available by the authors on request.
